# Supplementary material for: Broken force dispersal network in tip-links by the mutations at the Ca2+-binding residues induces hearing-loss
Source: Biochem J. 2019 Aug 30;476(16):2411–25. doi: 10.1042/BCJ20190453 (PMC6717114; doi:10.1042/BCJ20190453)
Supplement: Supplementary Figures and Tables [file BCJ-476-2411-s1.pdf]

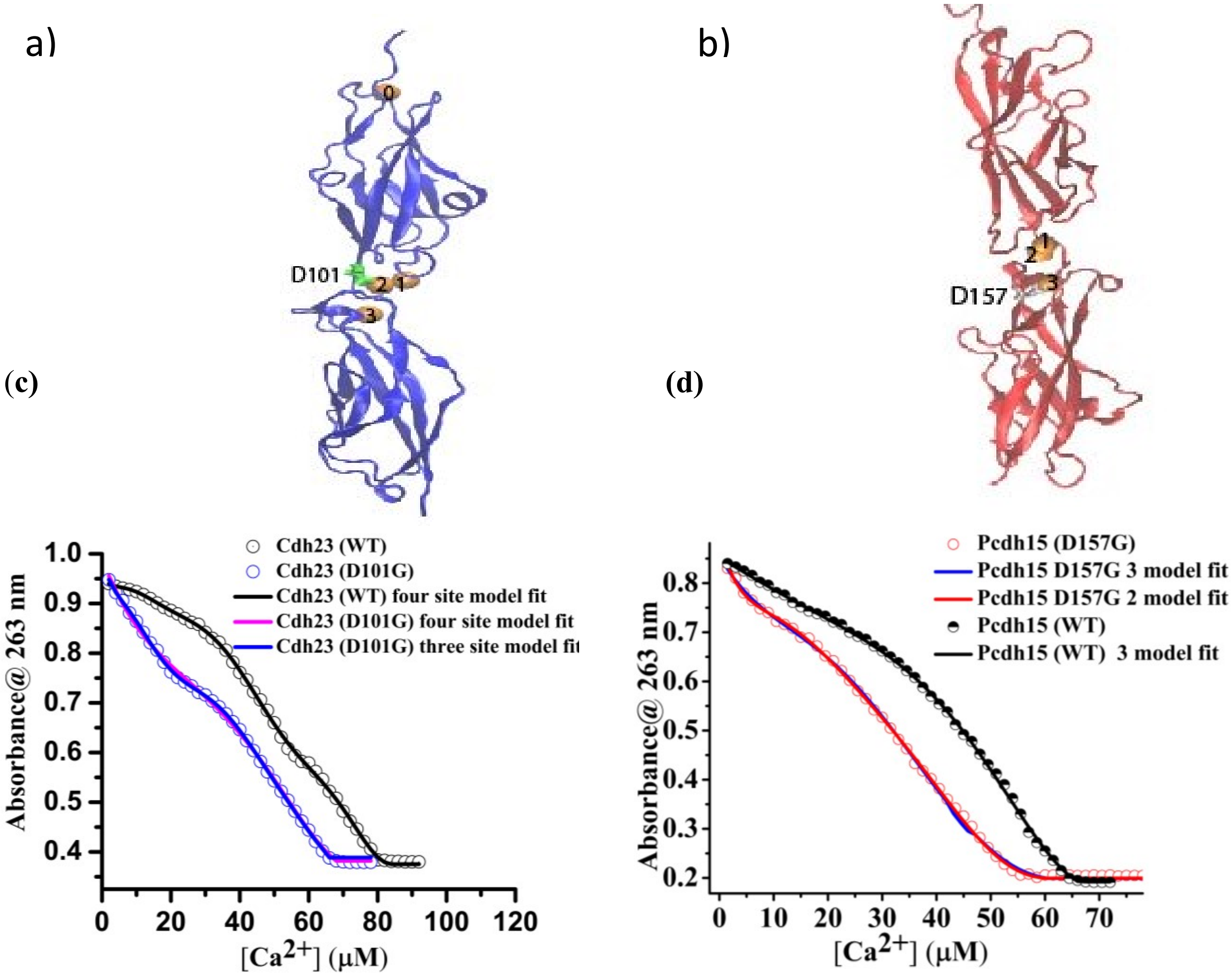

Figure S1: Determination of calcium binding constant using competitive chelator method (In support of Figure 1) (a) Structure of Cdh23(WT) marking the calcium ions. Aspartate at 101th position has been shown as green stick connected to 2nd calcium ion (b) Structure of Pcdh15(WT) with calcium ions (marked with number). Aspartate at 157th position has been showed in stick and connected to 3rd calcium ion (c) Gradual decrease in the absorbance of BAPTA at 263 nm with increasing Ca^{2+} concentration was monitored in presence of 25 μM of Cdh23 EC1-2(WT) (Black Circle), and in 25 μM of Cdh23 EC1-2(D101G) (Blue circles) independently (for further experimental procedures see text S1). Black line represents the four sites binding model fit for the WT. Blue solid line represents the fitting using three sites binding model and magenta line represents fitting using four sites binding model for the data correspond to Cdh23 EC1-2(D101G). The four binding site model for WT and three model fit for mutant was accepted from the F-test. (c) Gradual decrease in the absorbance of BAPTA at 263 nm with increasing Ca^{2+} concentration was monitored in presence of 25 μM of Pcdh15 EC1-2(WT) (Black Circle), and in 25 μM of Pcdh15 EC1-2(D157G) (red circles) independently. Black line represents the fitted curve obtained using three sites binding model for WT. Red and blue line represent the fitted curves obtained from three and two binding sites models respectively for Pcdh15 EC1-2(D157G). Three binding model was accepted for both WT and mutant from the F-test.

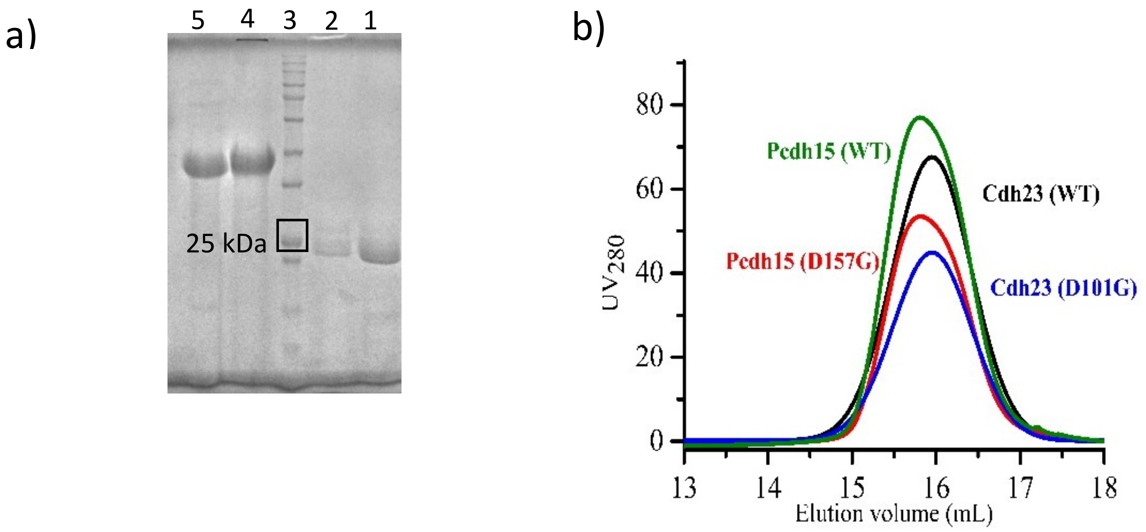

1- Cdh23 (WT)
2- Cdh23 (D101G)
3- Protein molecular weight marker
4- Pcdh15 (WT)
5- Pcdh15 (D157G)

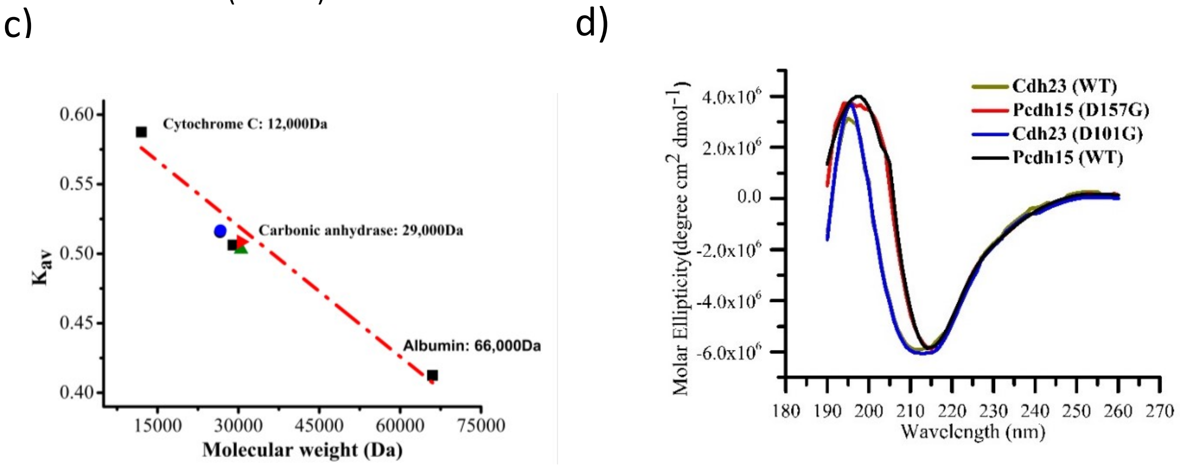

Figure S2: Purity and folding of the proteins(a) SDS-PAGE picture of all protein variants after size exclusion chromatography (b) Size exclusion chromatography (SEC) profile of the proteins performed at 50 μM Ca^{2+} using S200 GL column. (c) Putting the elution volumes in the calibration curve suggests the monomeric elution for all proteins in the monomeric forms.(d) Circular Dichroism (CD) experiments were carried out using Chirascan spectrophotometer at room temperature. Before CD experiment proteins were dialyzed in low salt buffer system (1 mM HEPES, 10 mM NaCl and 50 μM CaCl2) and finally CD spectras were collected of protein of concentration 15 μM. A quartz cell of path-length 1 mm was used for the measurement. Circular dichroism spectra of all the proteins obtained at 50 μM Ca^{2+} buffer showing the peak at 216 nm denoting the β -sheet rich structure of all the proteins.

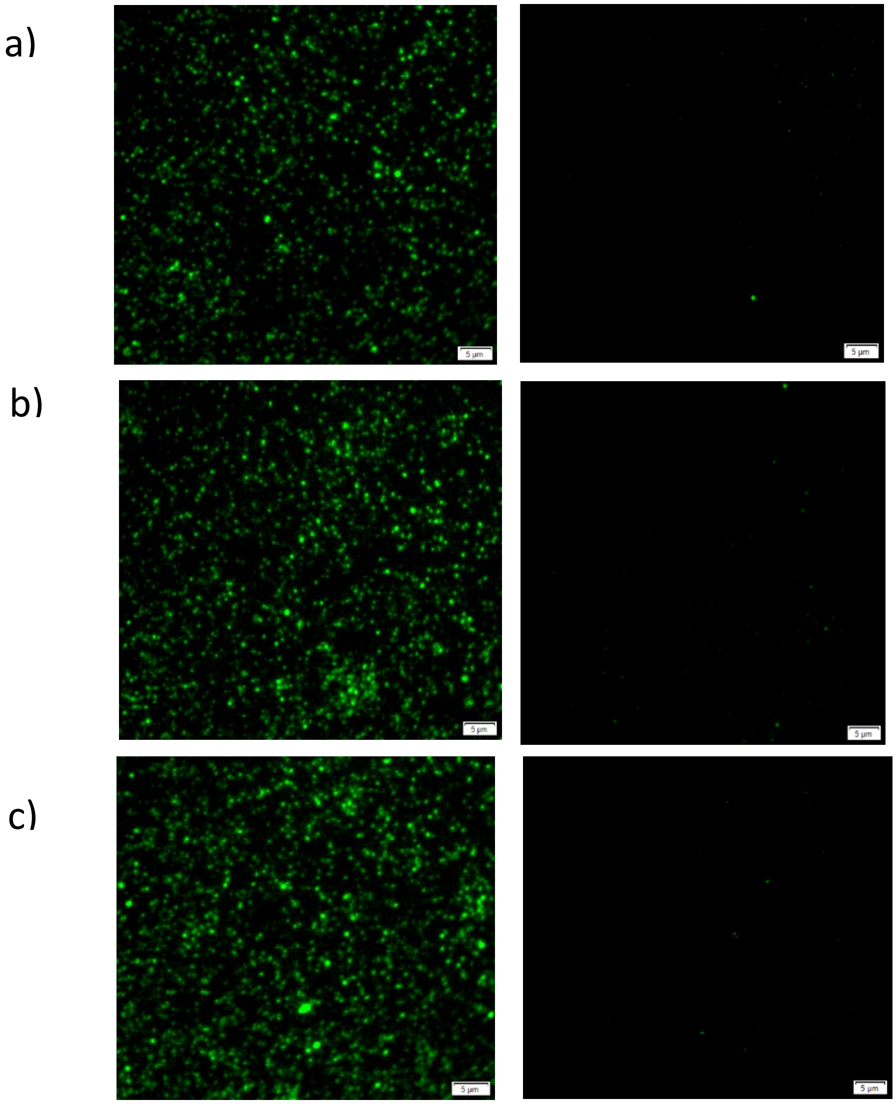

Figure S3: Single-molecule pull-down imagesdepicting the functionality of the protein(In support of Figure 1) (a) We covalently immobilized the C-terminal of unlabelled Cdh23(WT) on a glasssurface. Next, we incubated the surface with Cy3 labeled Pcdh15(WT) for 15 minsfollowed by thoroughwashing. We then visualized the surface under TIRF microscope by exciting with 532 nm laser at an exposure time of 300 ms and observed fluorescence signals (green spots)from theCy3-Pcdh15(WT) pulled down by Cdh23(WT). To monitor the specificity, we incubated the surface with 5mM EGTA buffer for half an hour under dark where EGTA chelates all Ca^{2+} out and monitored the signal again (right panel in a). We observed 92% drop in fluorescence signal, suggesting the interaction is Ca^{2+} dependent. (b) The pull-down of Cy3- Cdh23(D101G) by surface attachedPcdh15 EC1-2(WT) was performed, and subsequent loss of signal was monitored after EGTA wash (Right panel). (c) The pull-down of Cy3- PCdh15 (D157G) by surface attached Cdh23 EC1-2(WT) was performed, and subsequent loss of signal was monitored after EGTA wash (Right panel).

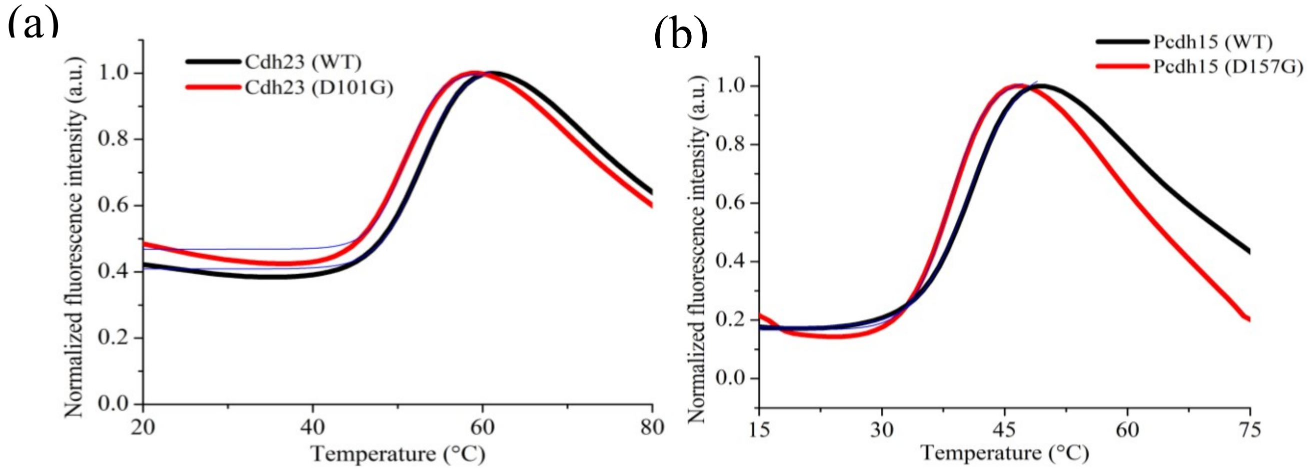

Figure S4: **Determination of melting temperature of Cdh23 and Pcdh15 variants using Differential scanning fluorimetry (DSF).** In order to check the stability of the protein upon mutation we carried out DSF measurements. Due to unfolding of protein hydrophobic patches open up. Sypro orange is one of the dyes of which quantum yield almost increase five times upon binding with hydrophobic patches. We used sypro orange as reporter dye of temperature induced unfolding of protein. Protein samples were diluted to 10 μM concentration in a buffer system containing 1 mM HEPES, 10 mM NaCl and 50 μM $CaCl_2$ of pH 7.6. Measurements have been performed in a RT PCR instrument. Excitation was done at 450-490 nm (FRET channel in a BioRad CFX 96 RT-PCR) and emission was collected at 570 nm of wavelength. Measurements were carried out by increasing temperature at the step of 1° C. Experiments were performed on a 96-well plate which was sealed to prevent the solvent evaporation. Finally, we calculated the melting temperature (T_m) of the proteins by fitting the sypro orange fluorescence emission intensity vs temperature curve obtained for each protein with the Boltzmann equation (equation 5). (a) Normalized sypro orange fluorescence emission intensity with temperature measured for Cdh23 (WT) (black) and Cdh23 (D101G) (red) with fitted curve (blue). (b) Normalized sypro orange fluorescence emission intensity varying with temperature measured for Pcdh15 (WT) (black) and Pcdh15 (D157G) (red) with fitted curve (blue). T_m obtained for Cdh23 WT, Cdh23 (D101G), Pcdh15 (WT), Pcdh15 (D157G) are 52.5 ± 0.4 °C, 49.6 ± 0.18 °C, 40.4 ± 0.41 °C, and 38.2 ± 0.37 °C respectively. We observed that both the mutations induced little destabilization to the proteins.

$$f_{calc} = f_{min} + \frac{f_{max} - f_{min}}{1 + e^{T_m - T}/_m} \quad (eq. 5)$$

f_{min}: Minimum fluorescence intensity, f_{max}: Maximum fluorescence intensity, T_m: Melting temperature, m: parameter to characterize width of transition.

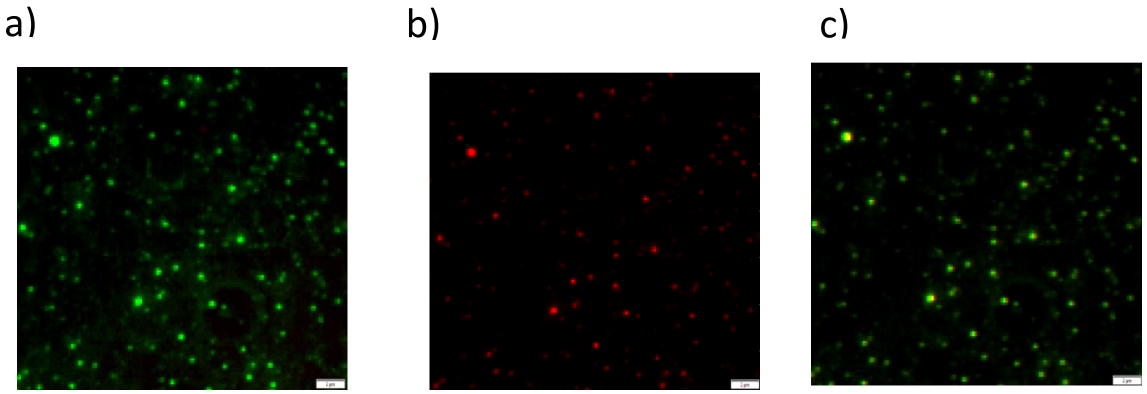

Figure S5: Representative TIRF images for the smFRET performed withCy3-Cdh23 EC1-2 on the surface incubated with Cy5-labeled Pcdh15 (In support of Figure 1) (a) Signal at the donor Cy3 channel,and (b) at the acceptor Cy5 channel. (c) Overlay of the Cy3 and Cy5 channel depicting the co-localization of the spots of both the channels. These images are representative of all three pairs, Cdh23(WT): Pcdh15(WT); Cdh23(WT):Pcdh15(D157G); and Cdh23(D101G):Pcdh15(WT), at varying Ca^{2+}.

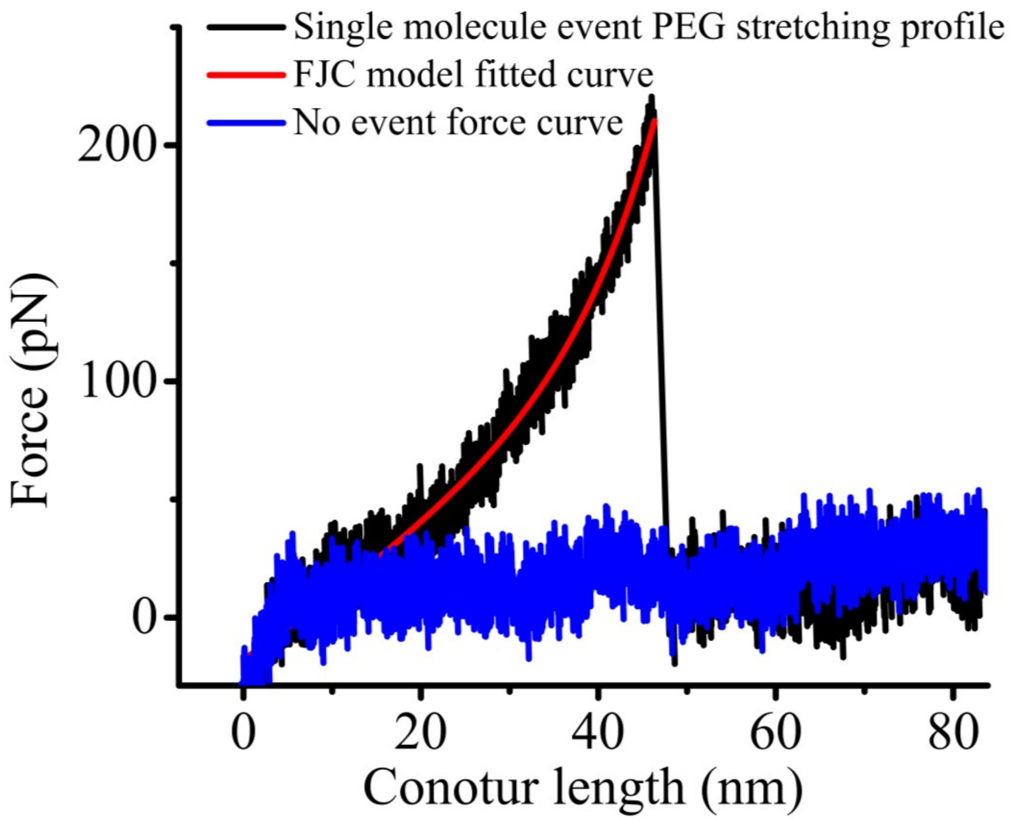

Figure S6: Characteristic single-molecule PEG stretching event with FJC model fit(In support of Figure 2) Black curve shows typical PEG stretching obtained during a single molecule force spectroscopy event along with FJC model fitting (red). Blue line depicts typical curve profile when there is no event.

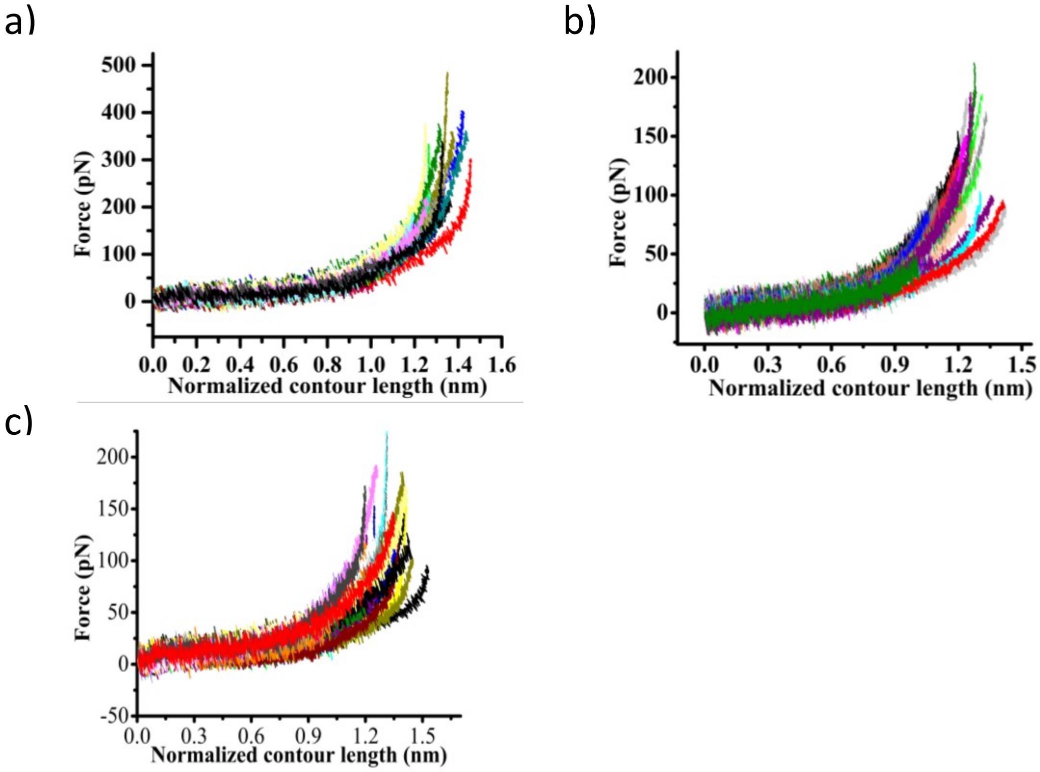

Figure S7. Selected experimental force-stretching curves for different protein pairs at pulling speed of 2000 nm.s^{-1}(In support of figure 2) (a) Cdh23 EC1-2 (WT) vs. Pcdh15 EC1-2(WT) interactions; (b) Cdh23 EC1-2 (D101G) vs. Pcdh15 EC1-2(WT) interactions; and (a) Cdh23 EC1-2 (WT) vs. Pcdh15 EC1-2(D157G) interactions. The curves are normalized by the extension (nm) corresponding to a constant force of 100 pN. The normalization protocol is described previously[13,14].

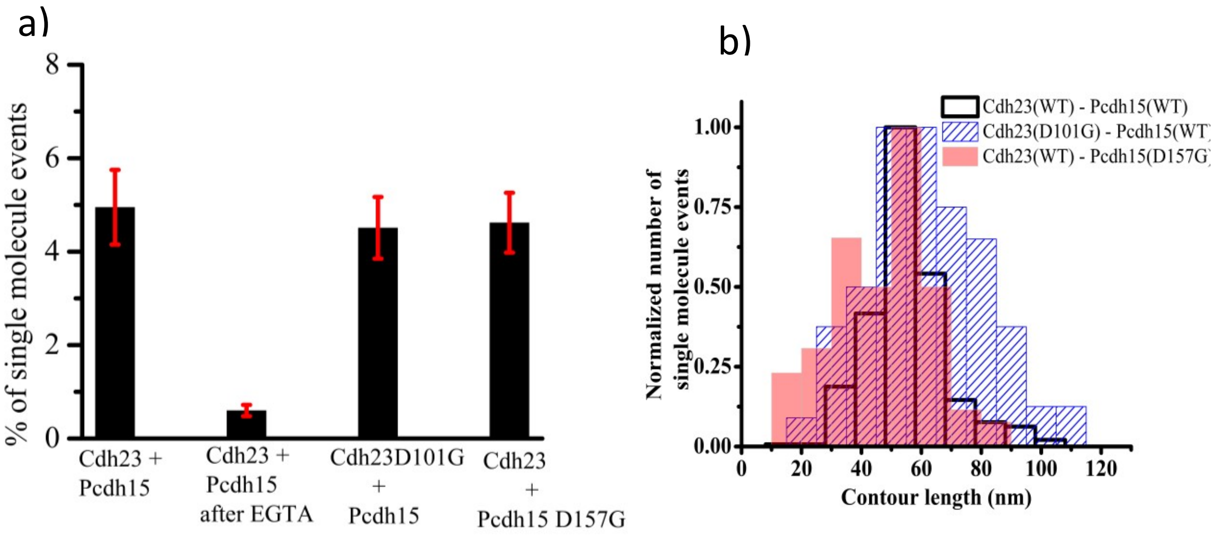

Figure S8: Percentage of single-molecule force-stretching events and the distribution of contour length of PEG (In support of Figure 2)(a) Percentage of single-molecule stretching events obtained for different protein pairs in presence and absence of Ca^{2+} are shown. (b) Contour-length of PEG is obtained from the fitting of each PEG stretching to WLC model as shown in S6. The distributions of contour-length for WT Cdh23 – WT Pcdh15 (black), Cdh23 D101G – WT Pcdh15 (blue) and WT Cdh23 – Pcdh15 D157G (red shaded) are similar and peaked at the theoretical estimate of two PEG stretching in series.

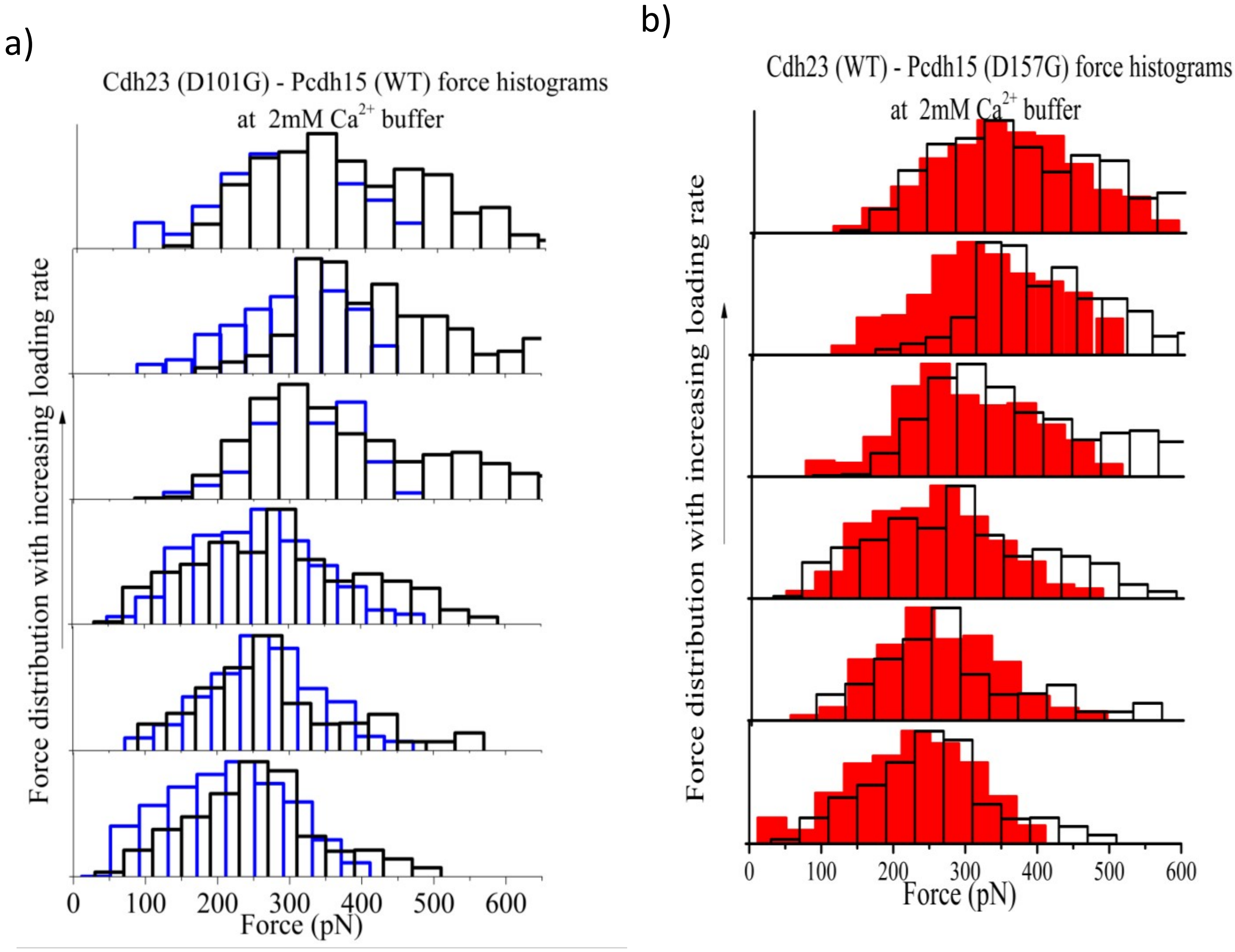

Figure S9: Force histogram of WT-mutant pairs at 2mM Ca^{2+} buffer.(In support of figure 2)(a) Force distributions obtained at different pulling velocities (200, 500, 10000, 2000, 30000, 50000 nms^{-1}) for Cdh23 (WT) – Pcdh15(WT) complexes (black) overlapped with Cdh23(D101G) – Pcdh15(WT) complex (blue). It shows that the force bearing property of the mutant complex gets restored at elevated calcium level. (b) Force distributions obtained at different pulling velocities (200, 500, 10000, 2000, 30000, 50000 nms^{-1}) for Cdh23 (WT) – Pcdh15 (WT) complexes (black) overlapped with Cdh23 (WT) – Pcdh15 (D157G) complex (red shaded). For this mutant also force sensing property becomes comparable to the WT complex at high calcium cocnentration.

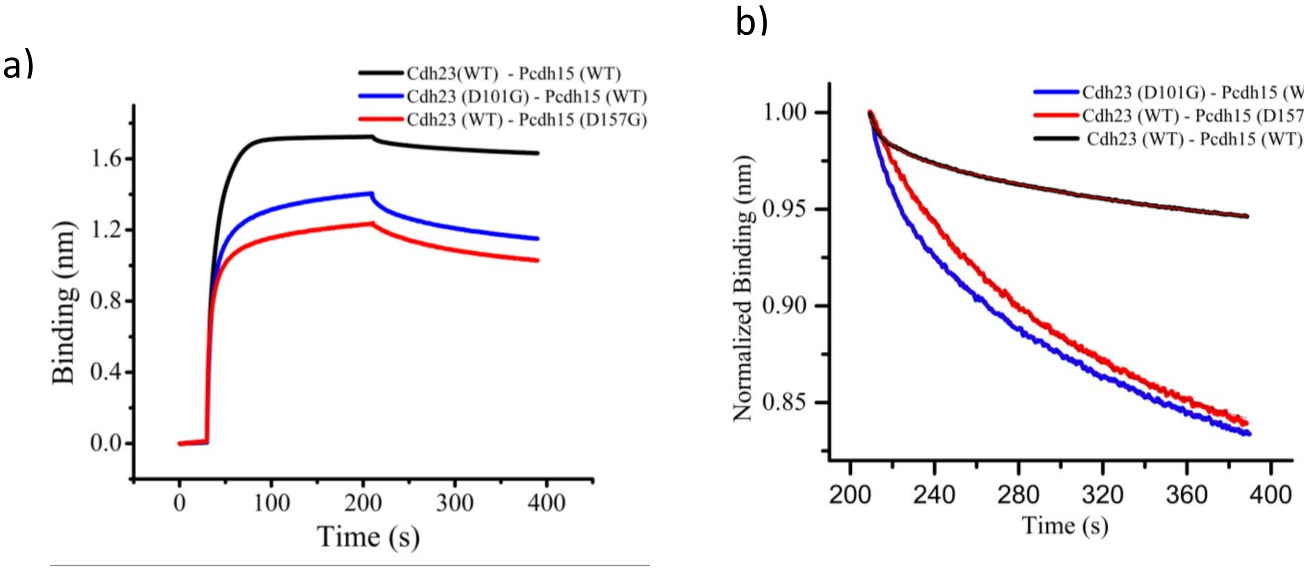

Figure S10: Bio-layer interferometry (BLI) to monitor the interactions between Cdh23(WT) Pcdh15(WT) and mutnat complexes (In support of Figure 2) We carried out Bio-layer interferometry (BLI) measurements using Amino-Propyl Silane (APS) sensor tip (Pall life science). We modified the APS tip with bifunctional NHS-PEG-Maleimide followed by polyglycine attachment as described above for smFRET and force spectroscopy. The density of the bifunctional PEG was maintained at 100 %. Next, the C-terminal of one of the protomers (analyte) was attached to the tip using sortagging, and the other protein was used as a ligand in 1 mL tube. Each cycle in the experiment consists of three steps. Step 1 is the baseline-correction where the sensor tip is immersed in the buffer alone for 30 s. Step II is the association step where the tip is dipped into the ligand proteins for 300 s. The third step is the dissociation step where the tip is again immersed in the buffer for 300 s. Data-fitting to 1:1 reaction model was done using the inbuilt fitting program of the Instrument.(a) The complete binding run for Cdh23(WT) - Pcdh15(WT), Cdh23(D101G) – Pcdh15(WT), and Cdh23(WT)-Pcdh15 (D157G) performed at 50 μM Ca^{2+} buffer are shown here. This depicts that the mutants are having lower on-rate and higher off-rate. (b) A normalized plot of the binding only during dissociation is highlighted here which clearly demostrates the lower off-rates for mutant-complexes. The rates were estimated from the 1:1 reaction modelfit to the data (mentioned in main-text).

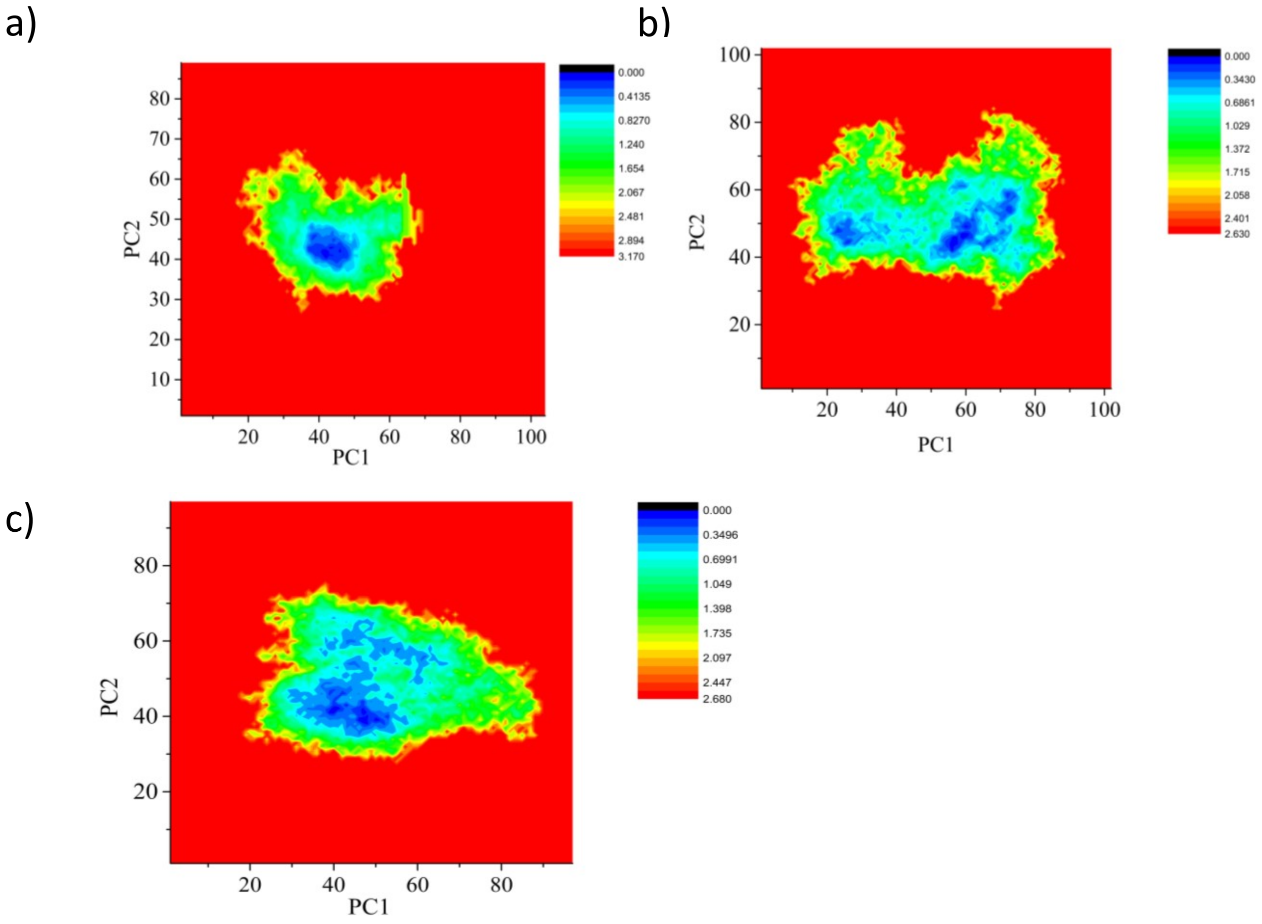

Figure S11: PCA based Free Energy landscape for the WT and mutant complexes (In support of Figure 3). The conformations visited during the three trajectories are projected on the Free Energy Landscape using the 1st and 2nd principal components as the reaction co-ordinates.(a) WT complex (with all Ca^{2+}), (b) Cdh23 (WT)-Pcdh15 (D157G) complex (3rd Ca^{2+}-removed), and (c) Cdh23 (D101G)-Pcdh15 (WT) mutant complex (2nd Ca^{2+}-removed). It is observed that the energy landscape of the WT complex has one well-defined energy basin, which is occupied by most of the conformations. However, the mutants have much higher conformational heterogeneity leading to broader occupancy of the energy landscape and the conformations are concentrated in two major energy basins with multiple small clusters inside them. This indicates that WT complex adopts a relatively rigid conformation but the complexes of the mutants are more flexible and exist is atleast two major conformations.

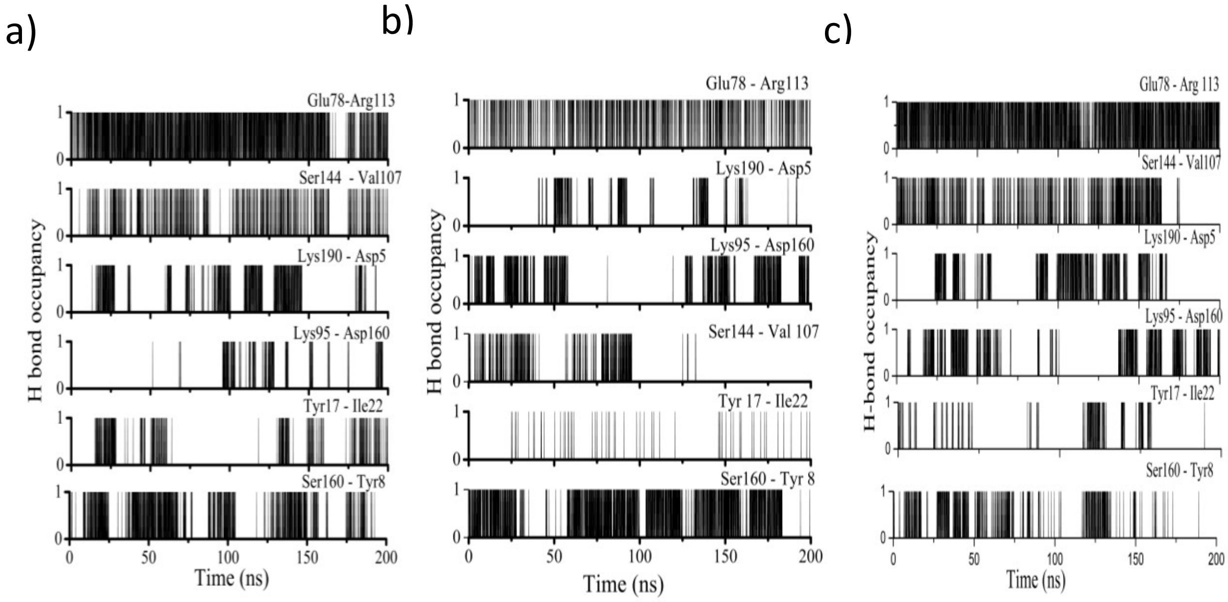

Figure S12: H-bond analysis of MD simulation(In support of figure 3)(a) Occupancy of various interprotein (between Cdh23 and Pcdh15) H-bond pairs during gaussian accelerated MD simulation of Cdh23 (WT) – Pcdh15 (WT) complex for 200 ns. (b) Occupancy of various interprotein H-bond pairs during gaussian accelerated MD simulation of Cdh23 (WT) – Pcdh15 (D157G) complex with one calcium ion removed for 200 ns. (c) Interprotein H-bond plots for Cdh23 (D101G) – Pcdh15 (WT)Complex simulation with one calcium removed performed for 200 ns.

(a)

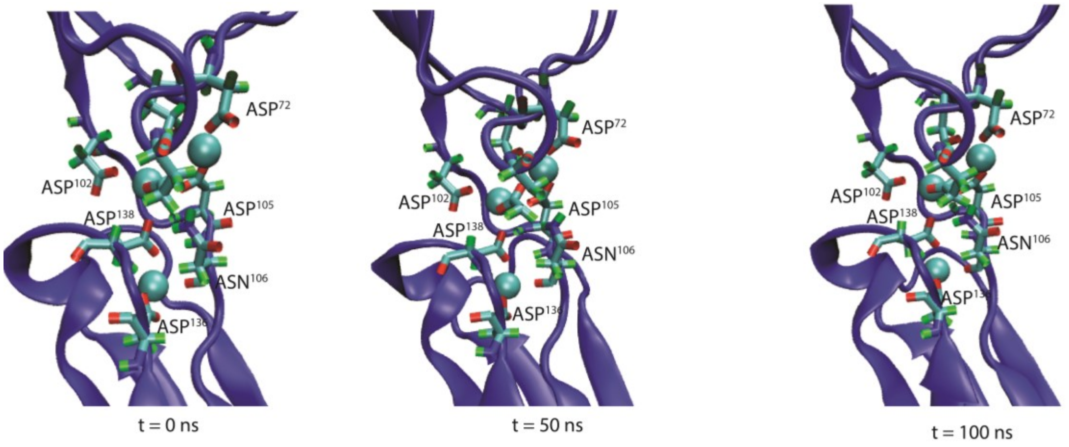
ASP^{72}
ASP^{102}
ASP^{138}
ASP^{105}
ASN^{106}
ASP^{136}
t = 0 ns
ASP^{72}
ASP^{102}
ASP^{138}
ASP^{105}
ASN^{106}
t = 50 ns
ASP^{72}
ASP^{102}
ASP^{138}
ASP^{105}
ASN^{106}
t = 100 ns

(b)

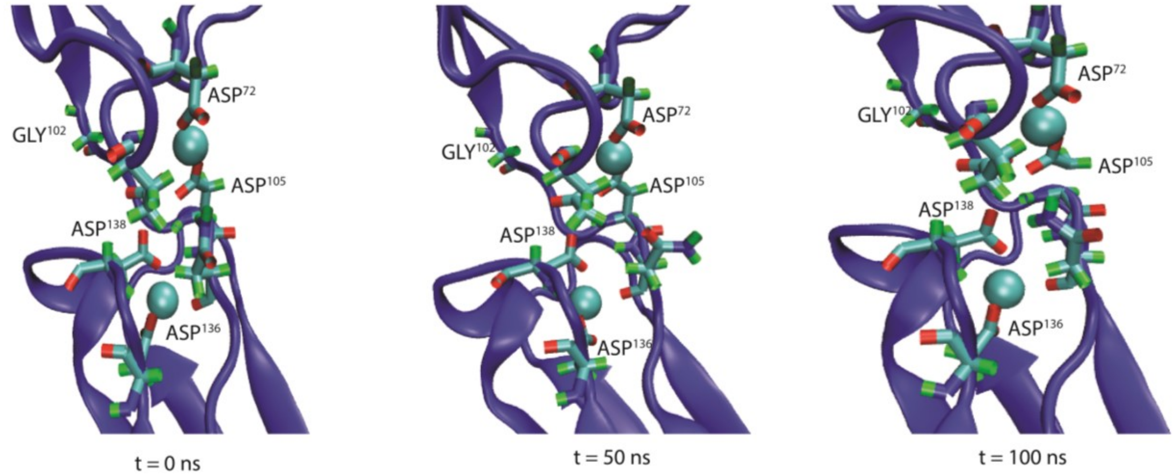
ASP^{72}
GLY^{102}
ASP^{105}
ASP^{138}
ASP^{136}
t = 0 ns
ASP^{72}
GLY^{102}
ASP^{105}
ASP^{138}
ASP^{136}
t = 50 ns
ASP^{72}
GLY^{102}
ASP^{105}
ASP^{138}
ASP^{136}
t = 100 ns

(c)

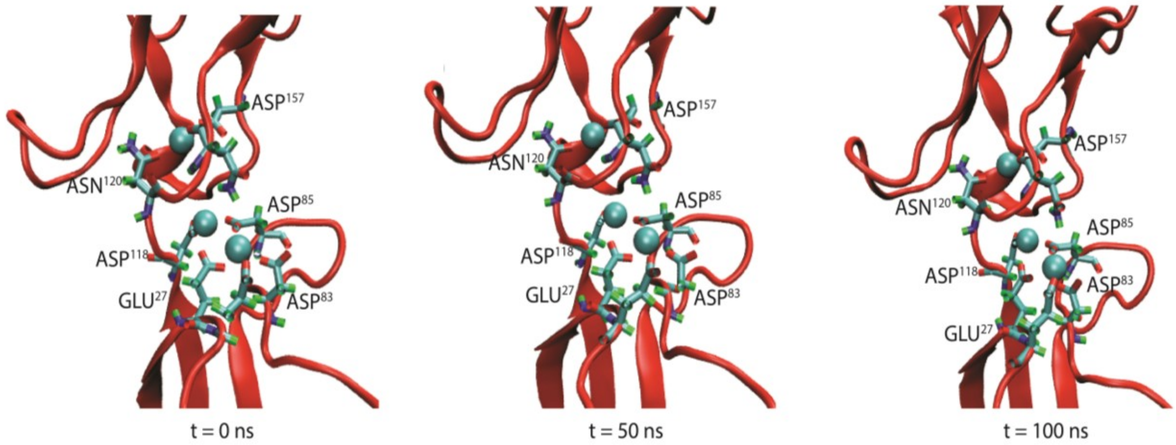
ASP^{157}
ASN^{120}
ASP^{85}
ASP^{118}
GLU^{27}
ASP^{83}
t = 0 ns
ASP^{157}
ASN^{120}
ASP^{85}
ASP^{118}
GLU^{27}
ASP^{83}
t = 50 ns
ASP^{157}
ASN^{120}
ASP^{85}
ASP^{118}
ASP^{83}
GLU^{27}
t = 100 ns

(d)

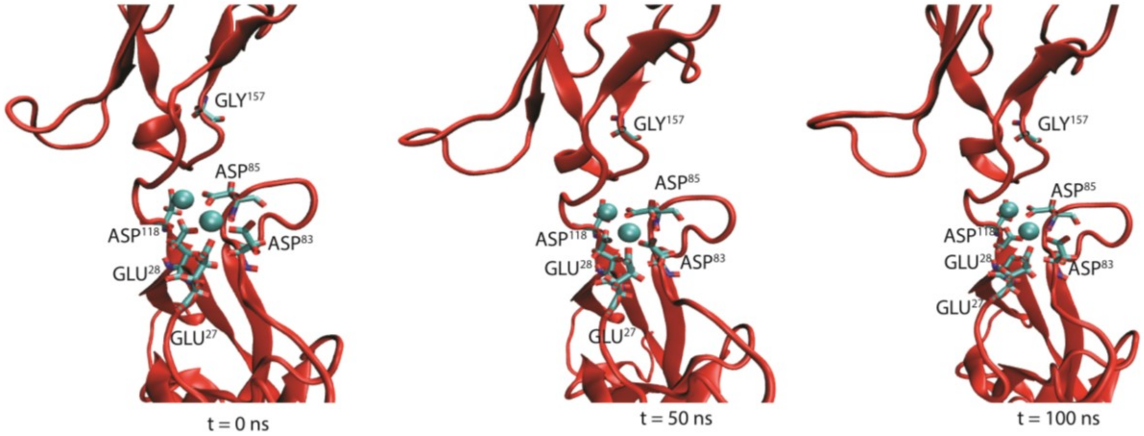

Figure S13: Snapshots of calcium coordination with residues at the linker region at the initial, middle and end of the 100 ns GAMD simulation for (a) Cdh23 (WT) three calcium coordination with residues at the linker region (b) Two calcium ions involving in coordination with negatively charged amino acids and presence of glycine mutation at 102nd position (c) Three calcium ions at the linker region in contact with negatively charged residues for Pcdh15 (WT) (d) Two calcium ions at the linker region for Pcdh15 (D157G) variant.

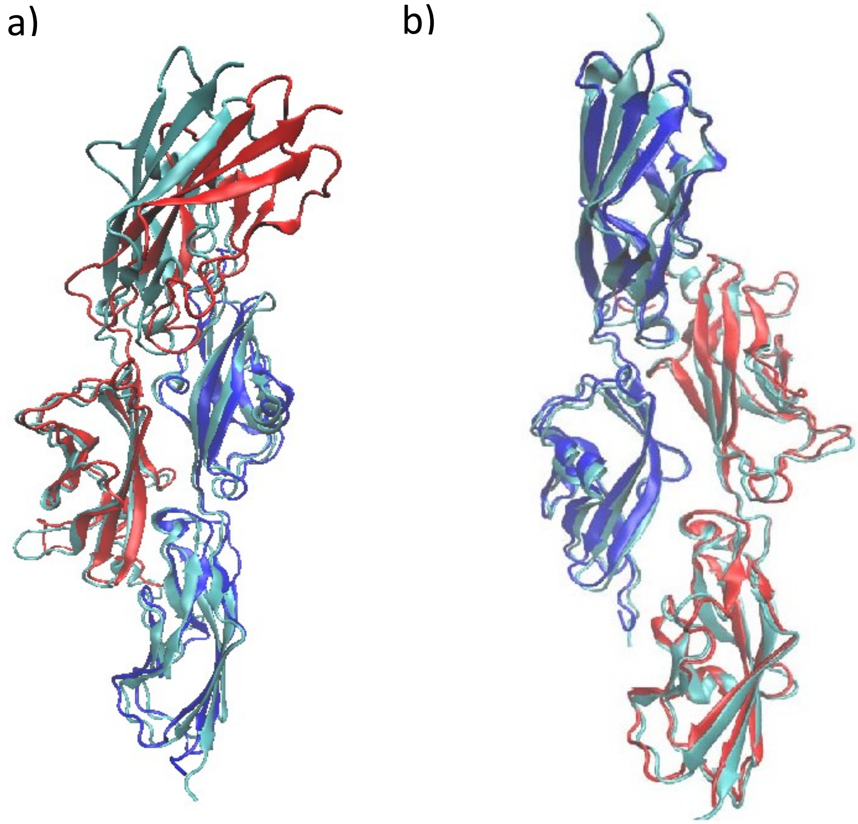

Figure S14: The most probable conformation of mutant complexes are overlapped with WT complex as obtained from cPCA (In support of figure 3). The most probable conformations were derived by averaging all the conformations obtained from the most populated cluster in the cPCA. (a) Overlap of WT with mutant-1 (Cdh23 (WT) – Pcdh15 (D157G) (red and blue, red denoting Pcdh15 and blue denoting Cdh23) and (b). Overlap of WT with mutant-2 (Cdh23 (D101G) – Pcdh15 (WT) (red and blue, red denoting Pcdh15 and blue denoting Cdh23). WT complex is denoted as cyan in both cases.

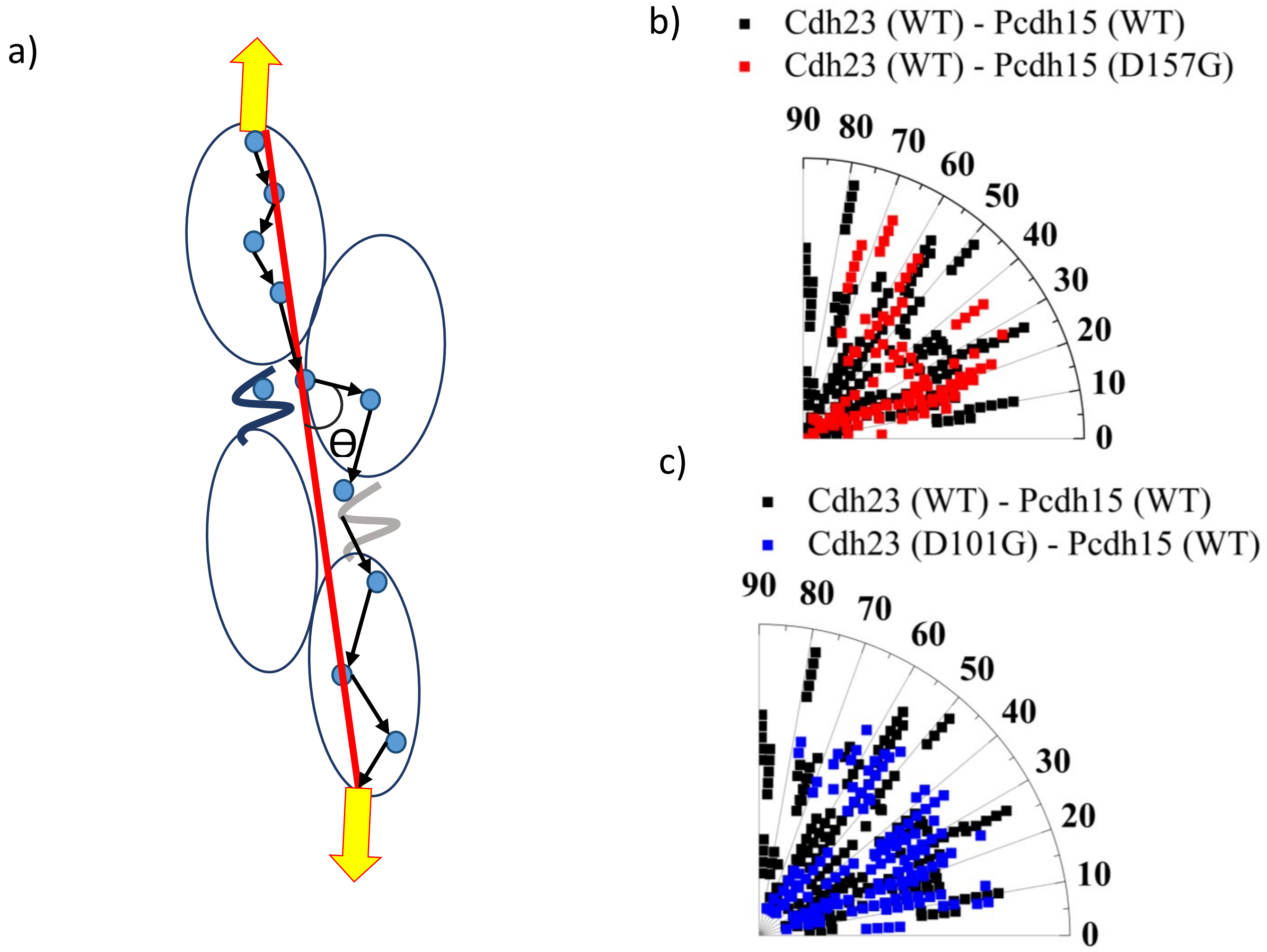

Figure S15: Distributions in the angle of force propagation vectors with the direction of pulling. **(In support of figure 3)** (a) Schematic description of the direction of pulling in SMD along with the angles measured between the edges connecting nodes (vectors, black arrows) and the pulling axis (red line) (Methods) for each suboptimal path. The black arrows describe the force propagation from one node to the next one. Nodes are represented in blue circles. (b) Comparison of the distributions of the angle of force propagation vectors for WT (black) and mutant-1 (red), and (c) for WT (black) and mutant-2 complex (blue). The vectors make smaller angles for both the mutant complexes than WT, indicating a smaller contribution in the perpendicular component of force-dispersal than the WT complex.

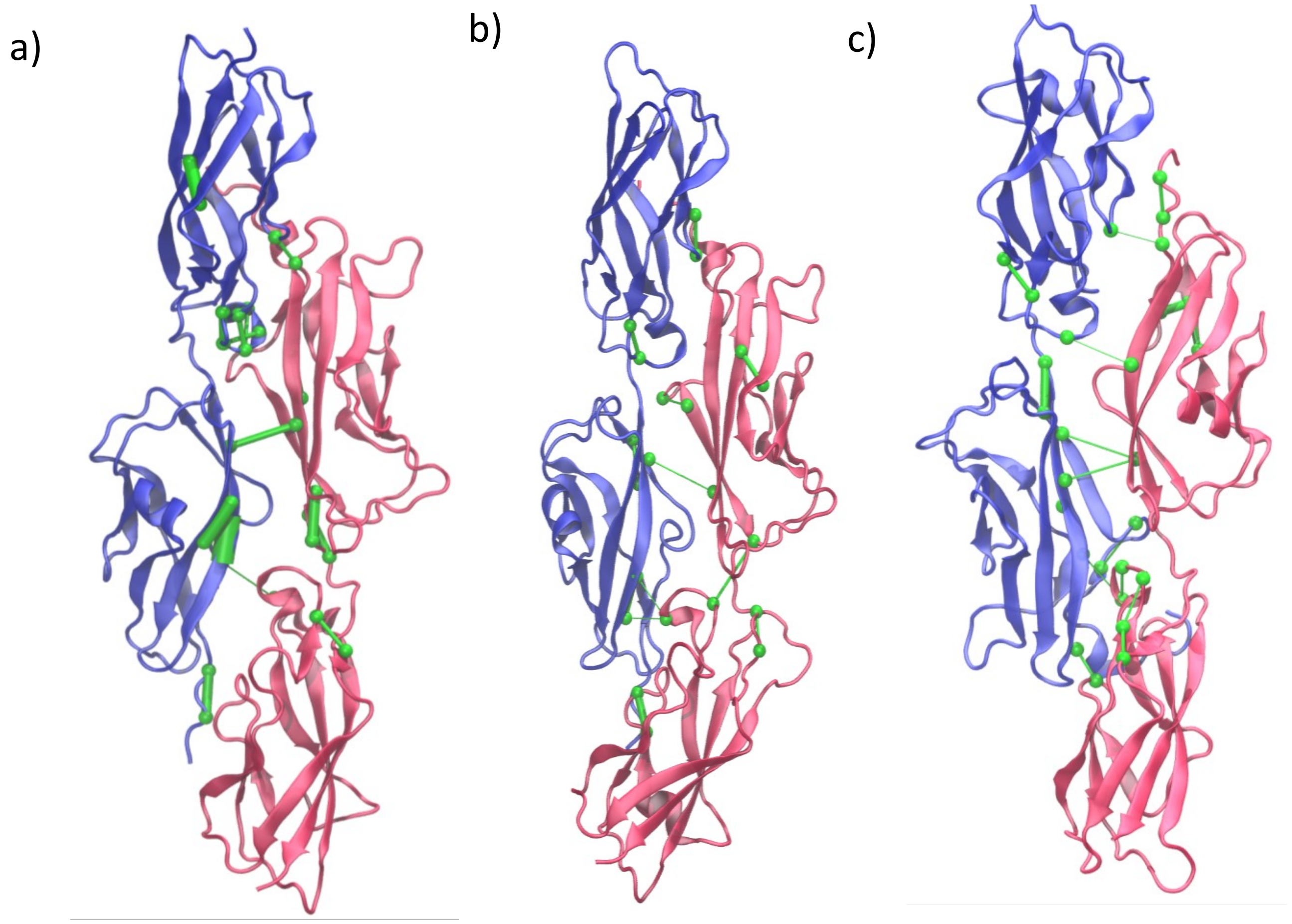

Figure S16: Depiction of critical nodes and edges along the force propagation pathway for WT and mutant complexes. (In support of figure 3) (a) Weighted critical nodes and edges for Cdh23 (WT) – Pcdh15 (WT) complex. (b) Cdh23 (WT) – Pcdh15 (D157G) complex. (c) Cdh23 (D101G) – Pcdh15 (WT) complex. Critical nodes were defined on the bases of their 'betweeness'. The correlation in the motion of the nodes was used to weight the edges and is depicted as the width of the edges. The WT complex is connected with more nodes and stronger (thicker) edges compared to D157G and D101G complexes indicating that the force-dispersal in WT complex is more than the mutants, thus making the WT more resilient to force and less vulnerable to force induced dissociation.

Text S1. Competitive chelator method for calcium binding constant determination

For the determination of calcium dissociation constant using competitive chelator method we used both quin 2 and BAPTA as calcium indicator. First, a stock 1 mM solution of the indicator was prepared in calcium free buffer. Protein solution was incubated with chelex resin for 2 hours to remove the calcium form the protein. Chelex was removed from the protein solution by centrifuging the solution at 2000 RPM for 10 minutes. Then equimolar amount (25 μM) of protein and indicator were mixed which will be used as titrant. Then calcium was added into the solution in a stepwise manner increasing the concentration at each step 3 μM and at each step absorption spectra was recorded from 200-500 nm wavelength using UV-Visible absorption spectrophotometer (Cary win UV-Visible spectrophotometer). From the spectra absorption change at 262 nm from each step was plotted with calcium concentration after each step. The curve was fitted with the equations 13 and 14 mentioned in reference no. 25 in various models i.e three binding sites, two binding sites, four binding sites model. Fitting was performed using MATLAB program written in-house. Earlier Sotomayor et, al determined the calcium binding affinity of Cdh23 (WT) using fluorescence based competitive chelator method and our results corroborated with their findings.

Table S1: Estimated Ca^{2+} dissociation constants of Cdh23 EC1-2 (WT) and Cdh23 EC1-2 (D101G) measured using competitive chelator method

Cdh23 (WT) (Four-sites model)	Cdh23 (D101G)	
	Four sites model	three sites model
2.8 ± 0.7<br>8.7 ± 3.2<br>34.2 ± 7.1<br>63.8 ± 11.4	3.6± 0.9<br>13.5 ± 3.7<br>99.2 ± 16.5<br>146.7 ± 23.8	5.7± 2.6<br>46.2± 9.1<br>98.2± 17.6

Table S2: Estimated Ca^{2+} dissociation constants of Pcdh15 EC1-2 (WT) and Pcdh15 EC1-2 (D157G) measured using competitive chelator method

PCdh15 (WT) (Three-sites model)	Pcdh15 (D157G)	
	Three sites model	Two sites model
34.6 ± 8.8<br>11.2 ± 6.5<br>42.5 ± 9.4	44.8± 7.4<br>16.3 ± 4.1<br>152.3 ± 11.9	46.5± 8.5<br>20.7± 4.7

Table S3: FRET efficiency and FRET distances for various complexes at different Ca^{2+}concentrations (from smFRET experiments)

[redacted]

Cdh23 (WT) – Pcdh15 (D157G) in 2 mM Ca^{2+} buffer	0.67±0.04	4.58±0.02

Table S4: Single molecule force-extension events obtained at different pulling velocities.

Cantilever Pulling velocity ($nm.s^{-1}$)	Number of events for Cdh23 (WT) – Pcdh15 (WT) out of 5625 measurements (Event-rate in %)	Number of events for Cdh23(D101G) – Pcdh15 (WT) out of 5625 measurements (Event-rate in %)	Number of events for Cdh23(WT) – Pcdh15 (D157G) out of 5625 measurements (Event-rate in %)
200	244 (4.3)	252(4.4)	277(4.9)
500	268 (4.7)	311(5.5)	246(4.3)
1000	232(4.1)	216(3.8)	293(5.2)
2000	327(5.8)	284(5.0)	213(3.7)
3000	287(5.1)	209(3.7)	302(5.3)
5000	323(5.7)	268(4.7)	241(4.2)

Table S5: Off-rate and on-rate for protein complexes obtained from BLI measurements

Protein pair	Off-rate (s^{-1})	On rate ($M^{-1}s^{-1}$)
Cdh23 (WT) – Pcdh15 (WT)	6.6E-04 ± 2.8E-05	5.1E+04 ± 2.4E+02
Cdh23 (D101G) – Pcdh15 (WT)	5.8E-03 ± 7.4E-04	9.9E+03 ± 5.9E+01
Cdh23 (WT) – Pcdh15 (D157G)	3.2E-03 ± 6.6E-04	6.5E+03 ± 2.3E+01

Table S6:Loading rates estimeated from each force-extension curves using model given by Ray et al[2] for all pairs of interaction

	Cdh23 (WT) – Pcdh15 (WT)		Cdh23 (D101G) – Pcdh15 (WT)		Cdh23 (WT) – Pcdh15 (D157G)	
Pulling velocity	Loading rate ($pN.s^{-1}$)	Standard error of mean	Loading rate ($pN.s^{-1}$)	Standard error of mean	Loading rate ($pN.s^{-1}$)	Standard error of mean
200	6632	56	5126	32	4165	40
500	16427	420	10478	52	11872	360
1000	38244	459	31024	120	36318	286
2000	58703	622	48211	223	47022	381
3000	92298	754	88204	475	81355	719
5000	147225	1419	112540	742	102114	833
